# Epidermal growth factor attenuates tubular necrosis following mercuric chloride damage by regeneration of indigenous, not bone marrow-derived cells

**DOI:** 10.1111/jcmm.12478

**Published:** 2014-11-11

**Authors:** Tzung-Hai Yen, Malcolm R Alison, Robert A Goodlad, William R Otto, Rosemary Jeffery, H Terence Cook, Nicholas A Wright, Richard Poulsom

**Affiliations:** a Department of Nephrology and Division of Clinical Toxicology, Chang Gung Memorial Hospital and College of Medicine, Chang Gung UniversityLinkou, Taiwan; b Histopathology Laboratory, Cancer Research UK, London Research InstituteLondon, UK; c Barts and The London School of Medicine and Dentistry, Queen Mary University of LondonLondon, UK; d Epithelial Stem Cell Group, Centre for Tumour Biology, Barts Cancer Institute, Barts and The London School of Medicine and Dentistry, Queen Mary University of LondonLondon, UK; e Molecular Pathology Facility, National Centre for Bowel Research and Surgical Innovation, Centre for Digestive Diseases, Blizard Institute, Barts and The London School of Medicine and Dentistry, Queen Mary University of LondonLondon, UK; f Department of Histopathology, Imperial CollegeLondon, UK

**Keywords:** epidermal growth factor, mercuric chloride, pegfilgrastim, acute tubular necrosis, bone marrow-derived cells, tubular regeneration

## Abstract

To assess effects of epidermal growth factor (EGF) and pegylated granulocyte colony-stimulating factor (P-GCSF; pegfilgrastim) administration on the cellular origin of renal tubular epithelium regenerating after acute kidney injury initiated by mercuric chloride (HgCl_2_). Female mice were irradiated and male whole bone marrow (BM) was transplanted into them. Six weeks later recipient mice were assigned to one of eight groups: control, P-GCSF+, EGF+, P-GCSF+EGF+, HgCl_2_, HgCl_2_+P-GCSF+, HgCl_2_+EGF+ and HgCl_2_+P-GCSF+EGF+. Following HgCl_2_, injection tubular injury scores increased and serum urea nitrogen levels reached uraemia after 3 days, but EGF-treated groups were resistant to this acute kidney injury. A four-in-one analytical technique for identification of cellular origin, tubular phenotype, basement membrane and S-phase status revealed that BM contributed 1% of proximal tubular epithelium in undamaged kidneys and 3% after HgCl_2_ damage, with no effects of exogenous EGF or P-GCSF. Only 0.5% proximal tubular cells were seen in S-phase in the undamaged group kidneys; this increased to 7–8% after HgCl_2_ damage and to 15% after addition of EGF. Most of the regenerating tubular epithelium originated from the indigenous pool. BM contributed up to 6.6% of the proximal tubular cells in S-phase after HgCl_2_ damage, but only to 3.3% after additional EGF. EGF administration attenuated tubular necrosis following HgCl_2_ damage, and the major cause of this protective effect was division of indigenous cells, whereas BM-derived cells were less responsive. P-GCSF did not influence damage or regeneration.

## Introduction

In 1989, Humes *et al*. published a pioneering study describing how infusion of epidermal growth factor (EGF) accelerated recovery from post-ischaemic acute tubular necrosis in a rat model 1. These findings prompted further studies of how EGF protects against, or accelerates recovery from, acute renal failure in various animal models. In a model of renal ischaemia-reperfusion injury, Norman *et al*. found that EGF treatment attenuated the rise in serum creatinine by 4 days after acute tubular necrosis and after 7 days, serum creatinine was again lower in animals receiving EGF 2. Both of those studies reported that infusion of EGF increased renal DNA synthesis as visualized by tritiated thymidine autoradiography. Coimbra *et al*. examined the effects of exogenous EGF during the recovery phase of HgCl_2_-induced acute renal failure 3. EGF caused greater levels of tritiated thymidine to become incorporated into renal proximal tubule cells, lower peak blood urea nitrogen (BUN) and serum creatinine levels and a 4-day reduction in the time required to return to near normal BUN and serum creatinine levels, as compared to non-EGF-treated animals. Morin *et al*. also observed a similar beneficial effect of exogenous EGF administration on the rate of tubular regeneration in their experimental model of gentamicin nephrotoxicity 4. Thus, exogenous EGF is effective at promoting recovery from a variety of acute kidney injury, even though a major source of EGF is the kidney itself.

We proposed in 2001 that the BM contributes to renal parenchyma regeneration 5. The presence of BM-derived cells was detected in both histologically normal mouse kidneys and in human transplanted kidneys with various causes of damage by using *in situ* hybridization to detect Y chromosomes together with markers of epithelial phenotype. The data indicate that BM stem cells contribute a low percentage of cells for both normal turnover of renal epithelia and regeneration after damage 5. In a subsequent study 6, female mice recipients of male whole BM were challenged with HgCl_2_ and the recovery of tubular damage scores and serum urea nitrogen (SUN) levels were assessed with or without erythropoietin (EPO) treatment. Confocal microscopy confirmed the tubular location of BM-derived cells and a four-in-one analytical technique (to identifying cell origin, tubular phenotype, tubular basement membranes and S-phase status) was developed to assess the relative contribution of BM to regenerative epithelium. BM-derivation of renal tubular epithelium increased from a baseline of 1.3–4.0% after HgCl_2_. EPO increased the haematocrit, but no other renoprotective effects were observed. We suggest that the principle underlying these observations is a natural influx of cells from BM to kidney that is stimulated by damage and assists regeneration and repair. EPO was unable to stimulate the process in this model of acute kidney injury, but other growth factors such as EGF may be able to do so, and this possibility merits investigation as a method of increasing the BM-derived population for regeneration or as a route for cell or gene therapy to help reduce the need for kidney transplants.

We report here a test of a strategy to increase the rate of regeneration of damaged kidneys in a way that could be considered for renal therapy. The questions we asked were: does administration of exogenous EGF improve recovery from acute tubular damage induced by HgCl_2_?; does EGF act equally on resident and BM-derived epithelium?; does a long-acting form of granulocyte colony-stimulating factor (pegylated-GCSF) increase numbers of BM-derived cells? and finally, is there an interaction between EGF and P-GCSF?

## Materials and methods

### Recombinant human EGF

Recombinant human EGF produced in *Saccharomyces cerevisiae* was a gift from Dr Jorge Berlanga-Acosta (Centre for Genetic Engineering and Biotechnology, Havana, Cuba). The purity of the EGF was 99% based on the results of high-pressure liquid chromatography and comprised 60% EGF_1–52_ (EGF protein lacking one terminal amino acid) and 40% EGF_1–51_. It has bioactivity equivalent to that of full-length EGF_1–53_ 7,8.

Prior to using EGF *in vivo*, we confirmed its bioactivity *in vitro* by assessing proliferation of human skin fibroblasts in DMEM as described 9. The effectiveness of 0, 5, 10 and 20 ng/ml EGF in 1% foetal calf serum (FCS) was assessed relative to the effects of 10% FCS using a PicoGreen fluorimetric DNA assay 10.

### BM adoptive transfer

All animal studies were performed under the UK Animals (Scientific Procedures) Act 1986. Before transplantation, C57BL6 female mice were given acidified drinking water (pH 2.8–3.2 with hydrochloric acid) for 1 week to prevent *Pseudomonas* growth. Six-week-old female recipient mice underwent whole body gamma-irradiation with 10 Gy in a divided dose 3 hrs apart to ablate their BM, followed immediately by tail vein injection of male whole BM (2 × 10^6^ cells). Thereafter, mice were given normal mouse feed and tap water *ad libitum*. There was no mortality after BM transplantation.

### Kidney damage and growth factor treatments

Six weeks after transplantation, 40 female recipient mice were randomly assigned to one of eight groups (Table1). In the control group, five were injected intraperitoneally (i.p.) with vehicle (0.2 ml of PBS). In the HgCl_2_ group, mice were given HgCl_2_ (203777, Sigma-Aldrich, Dorset, UK) at 3 mg/kg bodyweight (bw) i.p. This dose was chosen after a pilot study and was considered the sublethal dose enabling differentiation between control and treatment groups 11. Mice in the P-GCSF group received a single subcutaneous (s.c.) injection of 25 μg of P-GCSF (Neulasta®, Amgen, Abingdon, Oxon, UK) 12,13. Mice in the EGF group received 1 mg/kg bw EGF s.c. daily for 4 days. This dosage was based on our previous experience with the effects of EGF in rodents 14,15.

**Table 1:** Changes in serum urea nitrogen in female recipient mice after kidney damage and growth factor treatments (*N* = 40)

Mouse group	Serum urea nitrogen (mg/dl)
Control	13.58 ± 1.04
P-GCSF+	15.50 ± 1.10
EGF+	15.80 ± 1.40
P-GCSF+EGF+	14.98 ± 1.27
HgCl_2_+	158.10 ± 6.27*
HgCl_2_+P-GCSF+	155.52 ± 4.56*
HgCl_2_+EGF+	78.02 ± 3.08*,†
HgCl_2_+P-GCSF+EGF+	83.64 ± 2.42*

Pegylated granulocyte colony-stimulating factor P-GCSF, epidermal growth factor EGF, mercuric chloride HgCl_2_.

* *P* < 0.001 (compared to control group).

† *P* < 0.001 (compared to HgCl_2_ group). *N* = 5 per group.

### Radioactive thymidine injection, tissue harvesting and blood sampling

All recipient mice were killed 3 days after HgCl_2_ administration. This timing was based on our previous observation that tubular damage peaked 3 days following HgCl_2_ damage, followed by histological restitution at 14 days 6. To label cells undergoing DNA synthesis tritiated thymidine (TRK120, Amersham Biosciences, Chalfont St Giles, UK; 1 uCi/g bw i.p.) was given 1 hr prior to sacrifice. Kidneys, spleen and long bones were removed and fixed overnight in 10% neutral buffered formalin then transferred to 70% ethanol before being embedded in paraffin wax. Blood was obtained by cardiac puncture into a tube containing the lithium salt of heparinic acid as anticoagulant (Sarstedt) for determination of SUN using a diagnostic kit (Boehringer, Mannheim, Germany). No animals displayed dehydration or gastrointestinal bleeding at time of killing, either of which may cause a disproportional increment of SUN to creatinine.

### Assessing renal tissue injury

Sections (4 μm) were stained with Periodic acid–Schiff and haematoxylin & eosin. Analyses were performed blinded to the sample identity. In accordance with a procedure described previously 6,16, the degree of acute tubular necrosis was assessed by analysing three features of cell injury, tubular necrosis, tubular dilatation and cast formation, by point counting at 400× total magnification using a Weibel 1 graticule (Pyser SGI Ltd, Edenbridge, Kent, UK) with 50 ‘random’ points in each field. The number of points overlying the features of interest was determined. For each tissue section, 10 consecutive and non-overlapping fields (five cortex and five medulla) were scored. The results were presented as a percentage volume fraction (v/V × 100%) for each component, where v was the number of points recorded over the component and V was the total number of points scored (500).

### Immunohistochemical staining

Sections (4 μm) were dewaxed in xylene and endogenous peroxidases were blocked before sections were taken through graded alcohols to water and then to PBS 17,18. For antigen retrieval, sections were either subjected to microwaving (700 W) in 0.01 M citrate buffer at pH 6 for 10–20 min. or microwaving (700 W) in 2 mM ethylenediamminetetraacetic acid solution at pH 9.0 for 10–20 min.; or incubation with bovine trypsin [100 mg bovine trypsin (390414M; BDH Laboratory Supplies, Poole, UK), 100 mg calcium chloride, 100 ml distilled water, pH 7.8] at 37°C for 10–15 min. After washing with PBS, sections were incubated for 35 min. with either an antibody raised against CD45 (550539, BD Pharmingen, San Diego, CA, USA) at a 1/20 dilution or F4/80 (MCAP497; Serotec, Oxford, UK) at a 1/5 dilution or activated caspase 3 (AF835, R&D Systems, Abingdon, Oxon, UK) at a 1/100 dilution. For the second layer after PBS washing, sections were incubated with either biotinylated swine anti-rabbit (E353; Dako, Ely, UK) at a 1/500 dilution for 35 min. for rabbit antibodies, or rabbit anti-rat biotin (A0485; Dako) at a 1/100 dilution for rat antibodies. For the third layer after PBS washing, streptavidin-peroxidase (P397; Dako) at a 1/500 dilution was applied to sections for 35 min. Slides were developed in 3,3′-diaminobenzidine (D5637; Sigma-Aldrich) plus 0.3% hydrogen peroxide, counterstained in light haematoxylin, dehydrated and mounted in DPX-type mount.

### Four-in-one protocol

Sections were stained first for a tubular epithelial marker using lectin histochemistry, then for the Y chromosome using *in situ* hybridization (indirect method), plus for tubule basement membrane using periodic acid–Schiff (PAS), and finally cells actively synthesizing DNA (from ^3^H thymidine) were visualized by autoradiography 6.

### Lectin histochemistry

Four-micrometre sections were dewaxed, their endogenous peroxidases blocked (0.18% hydrogen peroxide in methanol) and then were taken through graded alcohols to PBS. Biotinylated lectins were used to stain proximal tubules [Phaseolus vulgaris leucoagglutinin (PHA-L); 1/1000; B-1115, Vector Laboratories, Orton Southgate, Peterborough, UK].

### *In situ* hybridization for Y chromosome

Lectin-stained sections were processed as described for direct detection, then were incubated with a peroxidase-conjugated anti-fluorescein antibody (11 426346 910; 1/250; Roche Diagnostics Ltd, Burgess Hill, UK) for 60 min. at room temperature, were washed, and Y signals were developed in 3,3′-diaminobenzidine (D5637; Sigma-Aldrich) plus 0.3% hydrogen peroxide. Slides were rinsed in PBS before PAS staining.

### PAS staining

Sections were oxidized with 1% aqueous periodic acid (BDH Laboratory Supplies) for 5 min., washed in water, then were incubated in Schiff's reagent (BDH Laboratory Supplies) before rinsing in distilled water for 10 min. each. A light haematoxylin counterstain was followed by dehydration through graded alcohols, air-drying and autoradiography.

### Autoradiography

Sections were dipped in LM-1 hypercoat emulsion (RPN40; Amersham Biosciences) at 45°C in a dark room under ‘safe light’ (902 filter) illumination. When dry, sections were stored in complete darkness at 4°C for 10–14 days then were developed, washed extensively, dehydrated, cleared and mounted in DePeX (BDH Laboratory Supplies).

### Fluorescence microscopy

Sections were examined using an Olympus BX61 epi-fluorescence microscope with SmartCaptureX software [Digital Scientific, Cambridge, UK (www.dsuk.biz)] to generate Red Green Blue images from multi-channel monochrome captures.

### Confocal microscopy

Laser scanning microscope system (LSM 510, Zeiss, Jena, Germany) with C-Apochromat 1.4NA ×40 water immersion objective lens was used to produce a Z-series of sequential scans imaging four channels: 4′,6-diamidine-2′-phenylindole dihydrochloride (DAPI: band pass 420–480 nm), fluorescein isothiocyanate (505–530 nm), red (560–615 nm) and far red (650 nm long pass) to visualize autofluorescent materials revealing structural landmarks within tissue sections. The Y-positive cells within tubules were easily identified by direct observation at an optical magnification of 400×. Examples of Y-positive cells were scanned at a higher magnification with 12-bit scan dimensions of 1024 × 1024 pixels, averaged eight times for each of 10–20 ‘optical sections’ of 0.4 μm in the Z-axis and archived using the Zeiss LSM 510 software package.

### Identification and counting of BM-derived tubular cells

To estimate the separate contributions of indigenous kidney (female) and BM-derived (male) cells to regeneration after renal injury, 1000 consecutively observed renal tubular epithelial cells per mouse were scored using a light- and dark-field microscope (200x magnification; Nikon Eclipse ME600, Tokyo, Japan). A proliferating BM-derived tubular cell was defined by the following criteria: positive for the tubular epithelial marker lectin, positive for a Y chromosome signal, observed within the Periodic acid–Schiff-stained tubular basement membrane, and exhibiting more than five silver grains overlying the nucleus after autoradiography. The 4-μm thickness of the sections enabled detection of the Y chromosome in 71% of tubule nuclei in male control kidneys. The counted values of Y-positive cells in the female recipients were divided by 0.71 to reflect the total donor-derived cell population.

### Statistics

Values presented in the text, tables and figures are given as means with the standard error of mean for the number of observations. Statistical analyses were performed with SPSS 11.0 for Mac (Chicago, IL, USA). The data were compared and analysed using anova test to compare the replicate means. The null hypothesis was rejected at a *P*-value of 0.05 or less.

## Results

### Confirmation of EGF bioactivity *in vitro* and *in vivo*

Epidermal growth factor increased the proliferation of cultured dermal fibroblasts (Fig. S1A). The DNA fluorescence yield of 10% FCS-treated cells (4731.50 ± 72.32 units, ***P < 0.001) compared with the 1% FCS-treated cells (3065.75 ± 52.30 units) served as a positive assay control. EGF supplementation of culture medium at both 10 ng/ml and the 20 ng/ml in 1% FCS increased DNA synthesis (3430.00 ± 109.33 units and 3463.50 ± 70.02 units respectively, *P < 0.05). The standard curve had a linear relationship between fluorescence and DNA concentration with a *R*^2^ > 0.95 (Fig. S1C).

The administered EGF was bioactive *in vivo*, as substantiated by a sharp increase in intestinal crypt cell proliferation (^3^H thymidine incorporation) in EGF-treated mice (Fig. S1D and E) compared with control mice (Fig. S1F and G).

### Confirmation of haematopoietic reconstitution

**Fig 1:**
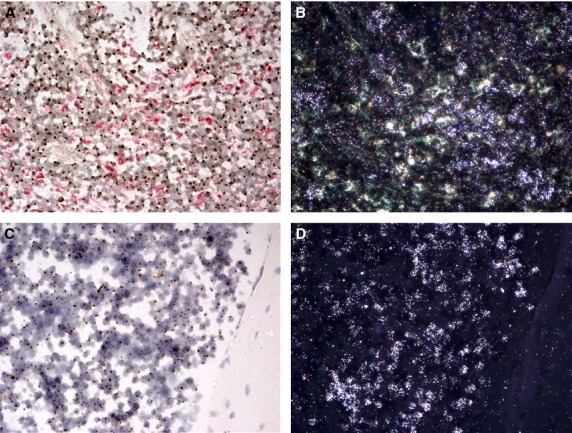
Confirmation of short-term haematopoietic reconstitution. Abundant Y-positive cells (brown nuclear dot) in the spleen (A and B) and BM (C and D) of a female recipient mouse indicate successful short-term haematopoietic reconstitution (bright field, A and C). Some of these Y-positive cells had incorporated ^3^H thymidine, detected by autoradiography as reflective silver grains under dark-field illumination (dark field, B and D).

The majority of cells in the spleen and BM were male in female recipient mice following lethal irradiation and male BM cell injection (Fig.1). Furthermore, some of these Y-positive cells had also incorporated ^3^H thymidine, indicating they were in S-phase (or undergoing substantial unscheduled DNA synthesis). The level of peripheral blood chimerism was not determined.

### Induction of acute kidney injury with HgCl_2_

**Fig 2:**
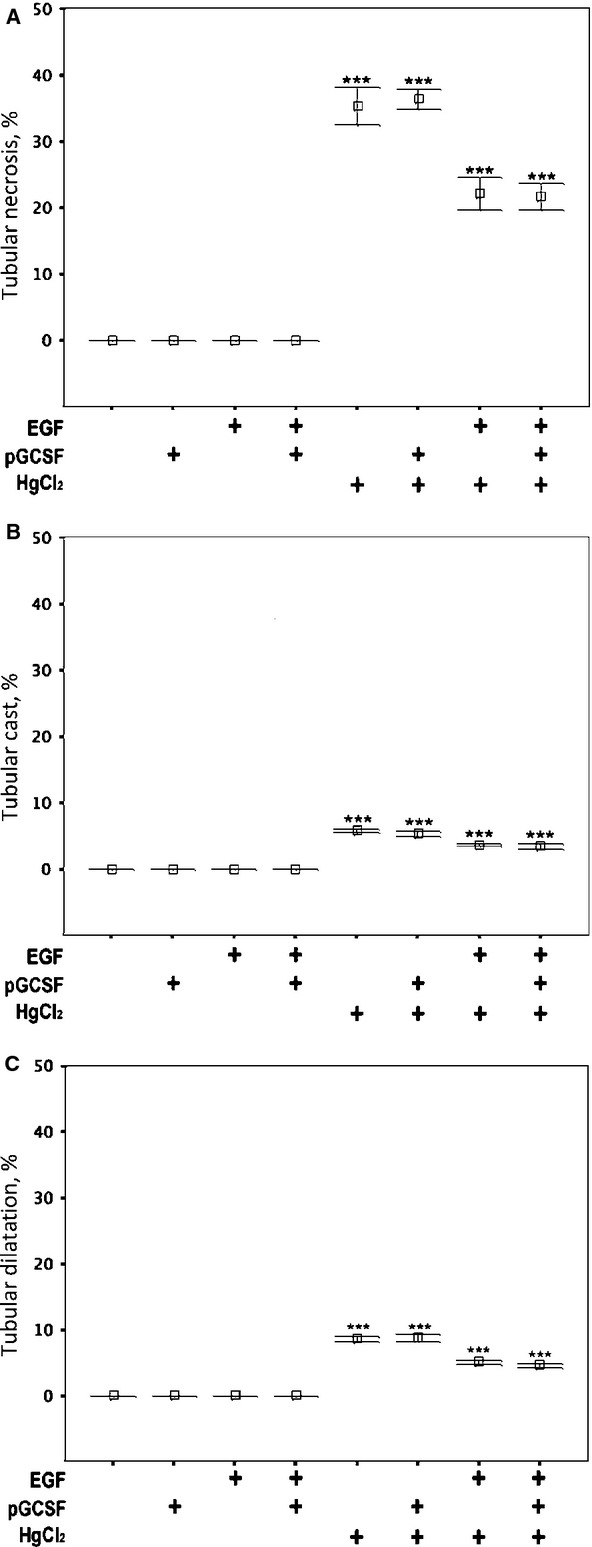
Attenuation of acute kidney injury by EGF and the absence of effects of P-GCSF. (A) Prevalence of tubular cell necrosis, (B) cast formation and (C) tubular dilatation after various treatments.

Three days after administration of HgCl_2_, the damaged kidneys were macroscopically enlarged, tense and pale. Microscopically, extensive necrosis of tubular epithelial cells was observed, with irregular spacing and a decreased number of cell nuclei. Some proximal tubules appeared dilated with flattened cells and attenuated brush borders. Some distal tubules were also dilated and exhibited flattened epithelial cells. Proteinaceous casts were found in many tubules. Tubular lumens contained many sloughed epithelial cells, leucocytes and cellular debris. There were apoptotic bodies (as revealed by active caspase 3 immunostaining) in the spleen (as positive internal control, data not shown), but not much found in the renal parenchyma after mercuric chloride damage. Therefore, most cell death because of mercury in this model was as a result of necrosis not apoptosis. Consistent with the strain of mouse used, no evidence of HgCl_2_-induced vasculitis was noted. Scores for features of tissue injury (Fig.2) were high after HgCl_2_ injection in contrast with the control group, which sustained no injury (*P* < 0.001). Injury scores were consistent with SUN measurements, and SUN was high and reached uraemic levels after HgCl_2_ injection (Table1).

### Attenuation of acute kidney injury by EGF

Epidermal growth factor was renoprotective in this model as demonstrated by the attenuated tubular injury scores (Fig.2) and the lowered SUN levels (Table1) in EGF-treated groups (HgCl_2_+EGF+ and HgCl_2_+P-GCSF+EGF+, *P* < 0.001) compared to HgCl_2_ alone (HgCl_2_+). Conversely, injected P-GCSF had no effect on these parameters.

### Renal infiltration of leucocytes after kidney damage and growth factor treatments

**Fig 3:**
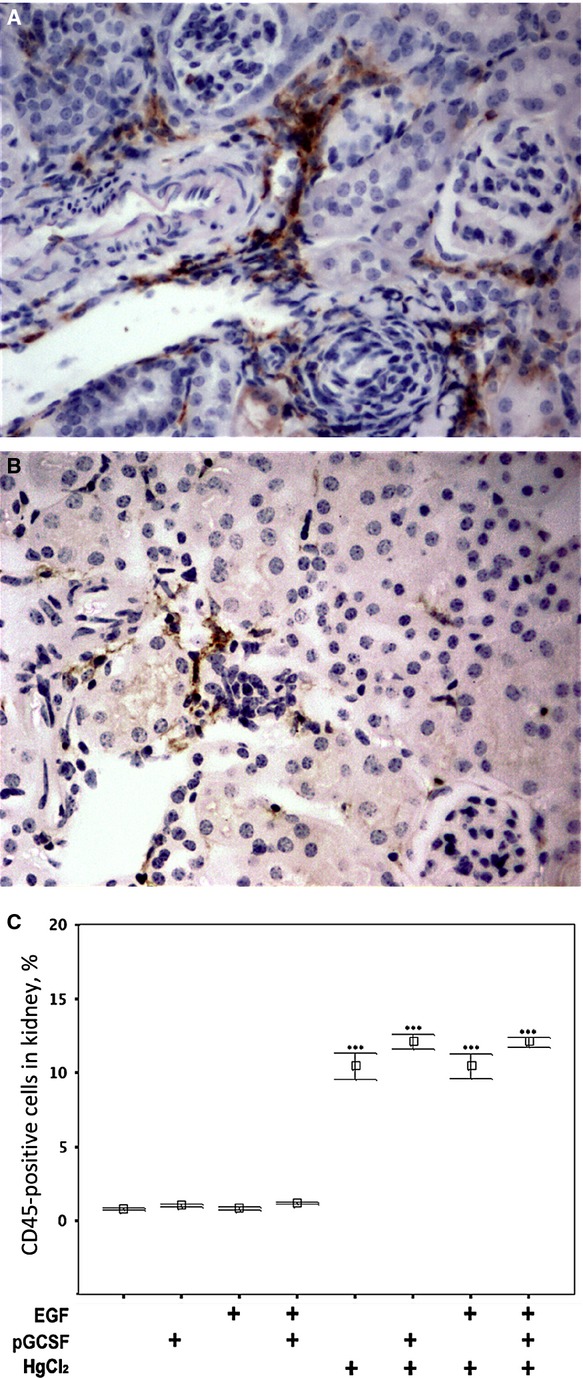
Renal infiltration of leucocytes and macrophages after kidney damage and EGF and P-GCSF treatments. Immunohistochemistry for (A) CD45 and (B) F4/80 reveals clusters of infiltrated leucocytes and macrophages respectively 4 days after induction of acute kidney injury in a female recipient of male BM. This mouse was treated with HgCl_2_ and EGF, but without P-GCSF treatment. (C) In mice with renal damage, P-GCSF increased the abundance of CD45-positive cells in the kidney (10.46 ± 0.44% *versus* 0.8 ± 0.04%, *P* < 0.001). EGF injections did not affect the abundance of CD45-positive cells.

Activated leucocytes are crucial in the pathogenesis of most kidney diseases from acute to chronic stages. In this study, levels of leucocyte infiltration after HgCl_2_ damage and growth factor treatments were monitored. Increased CD45-positive leucocyte recruitment to the kidney parenchyma was noted after HgCl_2_ damage (Fig.3A). Likewise, some limited recruitment of F4/80-positive macrophages to the damaged kidney was noted (Fig.3B). After damage, a significantly increased proportion of CD45-positive cells was noted in the kidney parenchyma after HgCl_2_ damage (10.46 ± 0.44%) in comparison with the control group (0.8 ± 0.04%) (Fig.3C). Moreover, mouse groups receiving P-GCSF injections displayed increased proportions of CD45-positive cells (12.1 ± 0.24% for HgCl_2_+P-GCSF+ and 12.1 ± 0.16% for HgCl_2_+P-GCSF+EGF+) compared to the HgCl_2_-only group (10.46 ± 0.44%), and the increase was statistically significant (*P* < 0.001). In contrast, the proportion of CD45-positive cells in mouse groups receiving EGF injections did not significantly differ from their counterparts (EGF+ *versus* control, HgCl_2_+EGF+ *versus* HgCl_2_+, *P* > 0.05).

### Confirmation of tubular location of BM-derived cells

**Fig 4:**
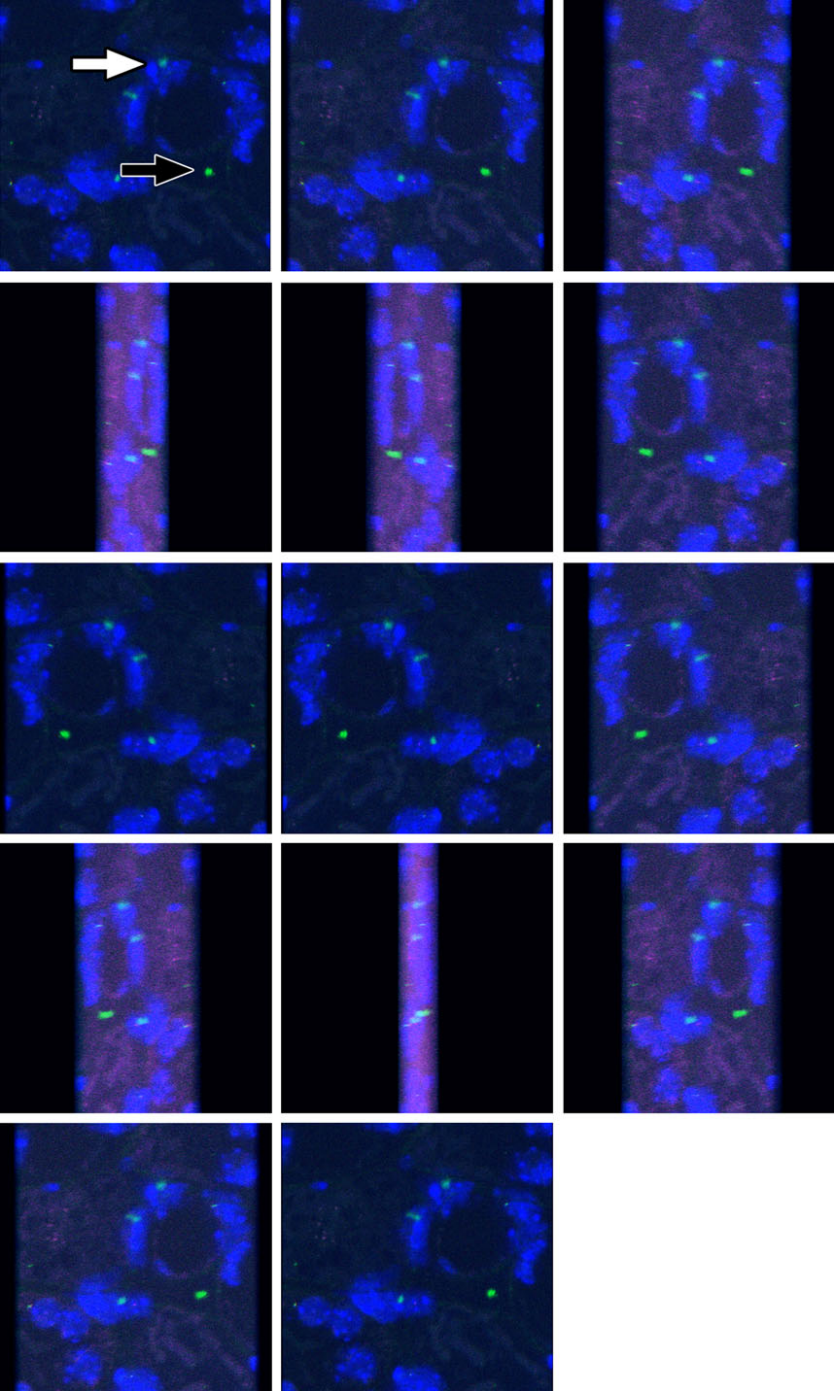
Four channel laser scanning confocal microscopy of a kidney section from a female mouse recipient of male bone marrow (BM), following mercuric chloride HgCl_2_ damage and EGF treatment. A gallery of images was generated from a projected Z-series to show how authentic fluorescence *in situ* hybridization signals for Y chromosomes (green) are present within cell nuclei (blue) (white arrow), whereas occasional green signals are artefactual (black arrow). The location of some BM-derived cells within tubular basement membranes is apparent from tissue architecture revealed by autofluorescence that has been captured in red and far-red channels. Authentic Y signals are always within the territory of a DAPI-stained nucleus.

To confirm the tubular location of BM-derived cells, kidney sections were subjected to fluorescent *in situ* hybridization for Y chromosomes and then examined under both fluorescent then laser scanning confocal microscopy. This allowed direct assessment that Y chromosome signals were actually within DAPI counterstained nuclei (Fig.4 white arrow) rather than being artefacts (Fig.4 black arrow).

### Cellular origin of the regenerating tubular epithelium

**Fig 5:**
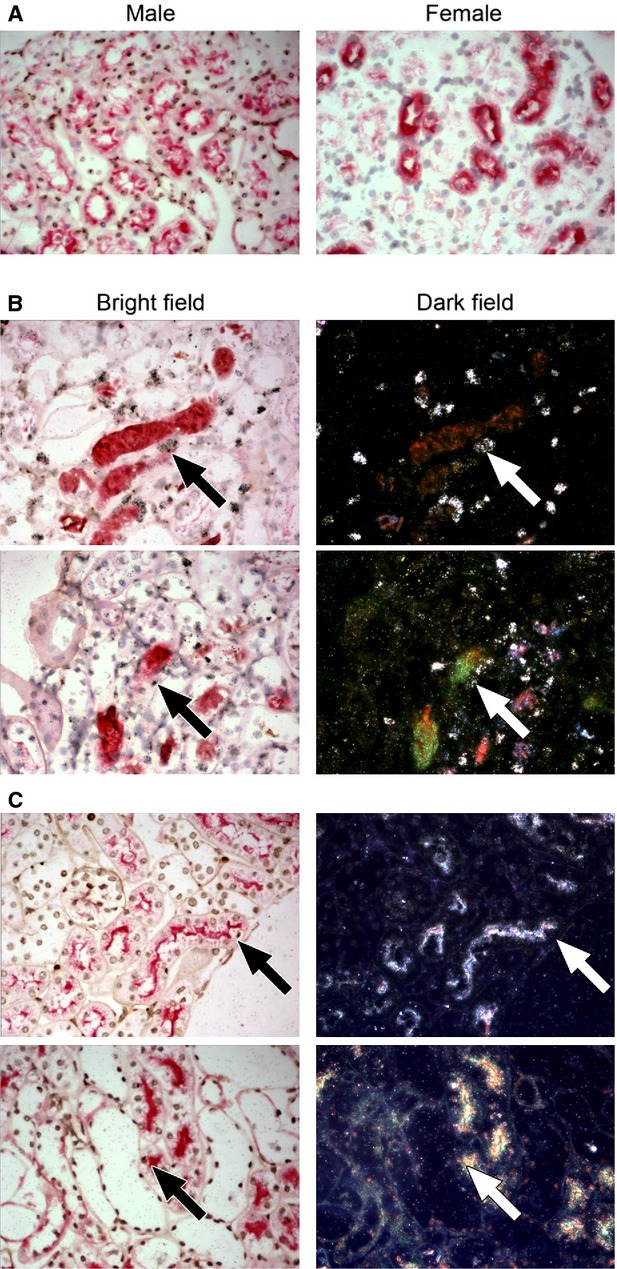
Detection of bone marrow (BM)-derived tubular epithelium in S-phase using the ‘four-in-one’ technique. (A) Control tissues stained with biotinylated *Phaseolus vulgaris* leucoagglutinin lectin (red) and indirect *in situ* hybridization for Y chromosomes (brown) showing co-localization of staining in the proximal tubule in male proximal tubules and no Y signals in female tissue. (B and C) Regions of kidney from female recipients of male BM that received HgCl_2_ damage and EGF treatment stained using the four-in-one protocol; these examples show BM-derived proximal tubule epithelial cells either (B) with or (C) without incorporation of ^3^H thymidine that reveals the BM-derived epithelial cell to be in S-phase. The dark-field panels allow autoradiographic silver grains to be seen as bright white dots.

**Fig 6:**
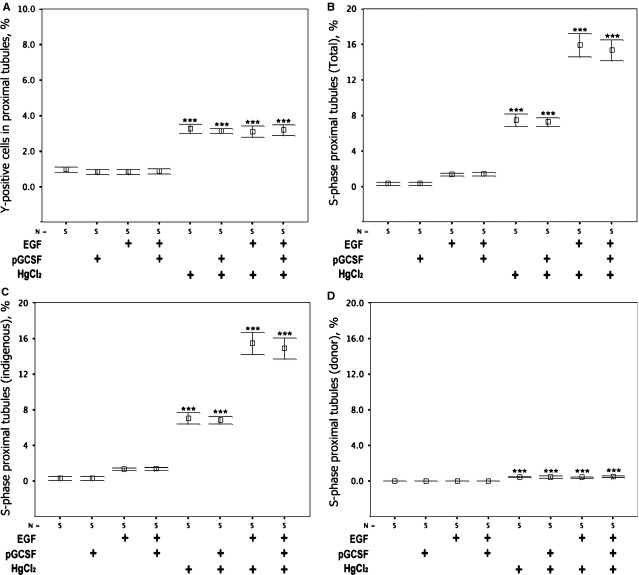
The effects of acute kidney injury, EGF and P-GCSF on the proportion of proximal tubular cell proliferation of BM-origin and on S-Phase status. (A) Acute kidney injury increased the proportion of proximal tubule cells derived from BM irrespective of treatment with EGF and/or P-GCSF, thus scoring of these cells was not affected by the influx of CD45 cells after P-GCSF. (B) The proportion of proximal tubule cells in S-phase was increased by acute kidney injury and EGF synergistically, whereas P-GCSF was without effect. (C) Indigenous proximal tubule cells responded to acute kidney injury and more so in combination with EGF, whereas P-GCSF had no effect on S-phase status. (D) A small but statistically significant increase occurred in the proportion of exogenous (BM-derived) proximal tubule cells in S-phase, with no effect of EGF or P-GCSF. Differences from corresponding control groups: ***P < 0.001. Means ± SEM, *n* = 5.

To establish the cellular origin of the EGF-mediated renal regeneration, sections of all kidneys were subjected to a combined ‘four-in-one’ analysing technique. This technique was designed to identify cell origin, tubular phenotype, reveal the tubular basement membrane and S-phase status of individual cells after renal damage (Fig.5). HgCl_2_ damage was found to increase significantly the proportion of proximal tubular cells that were BM-derived (Fig.6A) and in S-phase (Fig.6B) in all groups (*P* < 0.001). Regarding the cellular origin of these, the proximal tubular cells in S-phase, most were of indigenous kidney origin (Fig.6C, and few were derived from BM (Fig.6D). Exogenous EGF increased the proportion of proximal tubule cells in S-phase, particularly following acute kidney injury. P-GCSF did not affect S-phase status alone or in combination. BM contributed ∽1 in 15 of the S-phase proximal tubular cells after HgCl_2_ damage, but only ∽1 in 30 after additional EGF treatment.

## Discussion

We observed that tubular injury scores and SUN levels were high and reaching uraemic levels after HgCl_2_ injection, but as predicted EGF-treated groups were protected from such kidney damage. BM contributed ∽1% of proximal tubular epithelial cells in undamaged groups, increasing to ∽3% after HgCl_2_ damage. Neither EGF nor P-GCSF increased the abundance of BM-derived epithelial cells. The abundance of proximal tubular cells in S-phase was ∽0.5% in normal kidney, increasing to ∽7–8% after HgCl_2_ damage, and to ∽15% after exogenous EGF. The majority of the regenerating tubular epithelium was from the indigenous pool. BM contributed only to ∽1 in 15 of the proximal tubular cells in S-phase after HgCl_2_ damage, and to ∽1 in 30 after treatment with EGF. Thus, BM-derived cells might have inherent defects and were poorly responsive to EGF and this might place them at a disadvantage, causing them to be lost selectively as regeneration proceeds.

The cellular origin of newly differentiated tubular epithelium remains controversial 6. In several non-renal organs, local adult stem cells are recognized as the cell of origin for tissue replacement, such as haematopoietic system, skin and intestine 19. Many studies have suggested that exogenous BM cells, purified haematopoietic stem cells, or cultured mesenchymal stem cells (MSCs) may infiltrate mouse tissues and in particular, circumstances affect the course of renal damage 20. Results from acute kidney injury models, using glycerol 21, ischaemia-reperfusion 22–24, cisplatin 25, folic acid 26, HgCl_2_ 6 and genetically determined progressive failure in mouse models of Alport's syndrome 27,28 have been reported, and hypotheses have been generated to explain how cells from outside the kidney might modulate regenerative processes *via* transdifferentiation 29, cell fusion 29,30 or cytokine release 31. Furthermore, it has been recently reported in a murine model of cystinosis 32 that the therapeutic benefit of haematopoietic stem cell transplantation was independent of renal transdifferentiation, but instead, dependent on the level of bone marrow (BM) chimerism.

Our study tested the ability of a pegylated form of GCSF (a long-acting covalent conjugate of the recombinant methionyl human GCSF and monomethoxypolyethylene glycol) to mobilize BM and affect renal regeneration following HgCl_2_ damage. Both GCSF and P-GCSF are factors affecting haematopoietic cells by binding to specific cell surface receptors and thereby stimulating proliferation, differentiation, commitment and end-cell functional activation 33. Recent studies of cellular proliferation, receptor binding and neutrophil function demonstrate that GCSF and P-GCSF have similar mechanisms. As P-GCSF has low renal clearance and a prolonged half-life, renal dysfunction has no effect on the pharmacokinetics of P-GCSF; therefore, dose adjustment in animals with renal dysfunction is unnecessary. This growth factor is suited for experimental models of kidney failure where renal clearance tends to vary with time. No protective effect of P-GCSF was found in this model, which raised concerns whether the dosage was adequate, however the proportion of CD45-positive cells increased after HgCl_2_ and further increased after P-GCSF treatment.

In our study, generation of apparent ‘BM to kidney transdifferentiation’ was attempted using three strategies: HgCl_2_, lethal irradiation and P-GCSF. First, HgCl_2_ had a direct toxic effect on the S3 segment of the proximal tubule, causing moderate to severe acute tubular necrosis. Second, lethal irradiation enhanced the effect of the renal injury, either as a second injury or by inhibiting regeneration of surviving renal cells. Finally, P-GCSF further stimulated BM cells by recruiting circulating leucocytes to the damaged kidney. However, the abundance of BM-derived tubular cells was not affected by P-GCSF and this contrasts with the enhancement by GCSF of engraftment by BM-derived cells reported in murine models of liver disease 34.

Togel *et al*. examined whether increased circulation of CD34-positive cells, induced by their mobilization from the BM, would improve renal function and outcome in mice with ischaemic acute renal failure 35. Boosting peripheral CD34-positive cells (after cyclophosphamide and GCSF treatment) failed to exert any renoprotective effects but rather was associated with greatly increased severity of renal failure as well as increased mortality. Because identical ischaemic injury in neutropenic mice reduced renal insufficiency and significantly reduced mortality, it was deduced that adverse effects of pharmacological CD34-positive cells mobilization were primarily mediated by the concomitant induction of marked granulocytosis. Thus, high numbers of activated granulocytes apparently mask the potential renoprotective and positive survival effects of pluripotent haematopoietic stem cells, mediated by both their injurious renal and systemic actions. Similarly, there was no renoprotective effect of P-GCSF in this study.

Despite attenuation of renal damage, the percentage of S-phase proximal tubule cells in EGF rescued mice kidneys was only 16%. The reason was unclear, but possibly related to effect of whole body gamma-irradiation. In an earlier study 36, it was found that 2 days after ischaemia-reperfusion injury, 50.5% of outer medullary epithelial cells co-express Ki67 and red fluorescent protein, indicating that differentiated epithelial cells that survived injury undergo proliferative expansion. After repair was complete, 66.9% of epithelial cells had incorporated bromodeoxyuridine, compared to only 3.5% of cells in the uninjured kidney 36.

It has been proposed 37 that EGF could be used for *ex vivo* expansion of BM-derived MSCs, although species specific effects of EGF are reported: EGF stimulated the motility of rat and immortalized human BM-derived MSCs, but proliferation was inducible only in immortalized human BM-derived MSCs and not rat BM-derived MSCs. EGF caused robust phosphorylation of extracellular signal-regulated protein kinase (ERK) and protein kinase B/akt, but only minimal phosphorylation of EGFR and phospholipase C-gamma in rat BM-derived MSCs, whereas in the human BM-derived MSCs, these intermediaries were all strongly activated. EGF also induced robust ERK activation in primary porcine MSCs. EGF pre-treatment or co-treatment did not interfere with secondarily induced differentiation of either type of BM-MSC along adipogenic or osteogenic lineage, or rescue MSC from apoptosis induced by serum-deprivation. Taken together, these findings suggest that EGF may not be universally suited for *ex vivo* expansion and direction of BM-derived MSCs 37.

A potential concern over the use of EGF for driving renal regeneration is whether the long-term activation of EGF receptors on kidney cells would increase the chance of renal cell carcinoma 38. Many studies have documented increased overexpression of the EGF receptor (c-erbB1) and its ligands EGF and transforming growth factor-alpha in renal cell carcinoma 39. Furthermore, autocrine and paracrine signalling loops are associated with development and progression of renal cell carcinoma metastasis 40. Therefore, although strategies that activate EGF/EGF receptor may accelerate renal recovery, they may produce untoward proliferative effects if the activation is not carefully regulated. Preliminary data 41 from studies by our team in multiple intestinal neoplasia (Min) mice, that exhibit polyposis because of a genetic defect in the *Apc* gene, suggest that administration of EGF does not increase the number of polyps or degree of dysplasia, but does cause a 40% increase in polyp size in the proximal intestine specifically (*P* < 0.02). The remainder of the small intestine or colon, however, exhibits no such increase 41. No polyps were found in control mice given EGF and EGF did not initiate polyp formation in control or Min mice. However, as polyp size is an important determinant of subsequent risk for malignant change in human colon cancer, further studies are warranted of the effects of EGF.

## Conclusion

In summary, we confirm that treatment with exogenous EGF attenuated tubular necrosis following HgCl_2_ damage, and by comparing our data from a non-transplant setting (protective effect of EGF after HgCl_2_ damage) to a transplant model (following sex-mismatched BM transplantation), we have been able to assess the relative contribution of indigenous *versus* BM-derived cells to renal regeneration after damage. The action of EGF appears primarily to be to drive division of indigenous cells, whereas BM-derived cells – whether generated by fusion or not – were less responsive. P-GSCF had no renoprotective effects alone or in combination with EGF.

## Supporting Information

**Figure S1***In vitro* testing of epidermal growth factor (EGF) bioactivity.

## References

[b1] Humes HD, Cieslinski DA, Coimbra TM (1989). Epidermal growth factor enhances renal tubule cell regeneration and repair and accelerates the recovery of renal function in postischemic acute renal failure. J Clin Invest.

[b2] Norman J, Tsau YK, Bacay A (1990). Epidermal growth factor accelerates functional recovery from ischaemic acute tubular necrosis in the rat: role of the epidermal growth factor receptor. Clin Sci (Lond).

[b3] Coimbra TM, Cieslinski DA, Humes HD (1990). Epidermal growth factor accelerates renal repair in mercuric chloride nephrotoxicity. Am J Physiol.

[b4] Morin NJ, Laurent G, Nonclercq D (1992). Epidermal growth factor accelerates renal tissue repair in a model of gentamicin nephrotoxicity in rats. Am J Physiol.

[b5] Poulsom R, Forbes SJ, Hodivala-Dilke K (2001). Bone marrow contributes to renal parenchymal turnover and regeneration. J Pathol.

[b6] Yen TH, Alison MR, Cook HT (2007). The cellular origin and proliferative status of regenerating renal parenchyma after mercuric chloride damage and erythropoietin treatment. Cell Prolif.

[b7] Sinha A, Nightingale J, West KP (2003). Epidermal growth factor enemas with oral mesalamine for mild-to-moderate left-sided ulcerative colitis or proctitis. N Engl J Med.

[b8] Calnan DP, Fagbemi A, Berlanga-Acosta J (2000). Potency and stability of C terminal truncated human epidermal growth factor. Gut.

[b9] Otto WR, Nanchahal J, Lu QL (1995). Survival of allogeneic cells in cultured organotypic skin grafts. Plast Reconstr Surg.

[b10] Otto WR (2005). Fluorimetric DNA assay of cell number. Methods Mol Biol.

[b11] Hultman P, Enestrom S (1986). Localization of mercury in the kidney during experimental acute tubular necrosis studied by the cytochemical Silver Amplification method. Br J Exp Pathol.

[b12] Morris ES, MacDonald KP, Rowe V (2004). Donor treatment with pegylated G-CSF augments the generation of IL-10-producing regulatory T cells and promotes transplantation tolerance. Blood.

[b13] Morris ES, MacDonald KP, Rowe V (2005). NKT cell-dependent leukemia eradication following stem cell mobilization with potent G-CSF analogs. J Clin Invest.

[b14] Berlanga-Acosta J, Playford RJ, Mandir N (2001). Gastrointestinal cell proliferation and crypt fission are separate but complementary means of increasing tissue mass following infusion of epidermal growth factor in rats. Gut.

[b15] Goodlad RA, Raja KB, Peters TJ (1991). Effects of urogastrone-epidermal growth factor on intestinal brush border enzymes and mitotic activity. Gut.

[b16] Turnberg D, Botto M, Lewis M (2004). CD59a deficiency exacerbates ischemia-reperfusion injury in mice. Am J Pathol.

[b17] Yen TH, Chen Y, Fu JF (2010). Proliferation of myofibroblasts in the stroma of renal oncocytoma. Cell Prolif.

[b18] Yen TH, Yang HY, Yeh YH (2013). Aliskiren attenuates proteinuria in mice with lupus nephritis by a blood pressure-independent mechanism. Lupus.

[b19] Yen TH, Wright NA (2006). The gastrointestinal tract stem cell niche. Stem Cell Rev.

[b20] Poulsom R (2007). CD44 and hyaluronan help mesenchymal stem cells move to a neighborhood in need of regeneration. Kidney Int.

[b21] Herrera MB, Bussolati B, Bruno S (2004). Mesenchymal stem cells contribute to the renal repair of acute tubular epithelial injury. Int J Mol Med.

[b22] Kale S, Karihaloo A, Clark PR (2003). Bone marrow stem cells contribute to repair of the ischemically injured renal tubule. J Clin Invest.

[b23] Behr L, Hekmati M, Fromont G (2007). Intra renal arterial injection of autologous mesenchymal stem cells in an ovine model in the postischemic kidney. Nephron Physiol.

[b24] Lin F, Moran A, Igarashi P (2005). Intrarenal cells, not bone marrow-derived cells, are the major source for regeneration in postischemic kidney. J Clin Invest.

[b25] Morigi M, Imberti B, Zoja C (2004). Mesenchymal stem cells are renotropic, helping to repair the kidney and improve function in acute renal failure. J Am Soc Nephrol.

[b26] Fang TC, Alison MR, Cook HT (2005). Proliferation of bone marrow-derived cells contributes to regeneration after folic acid-induced acute tubular injury. J Am Soc Nephrol.

[b27] Ninichuk V, Gross O, Segerer S (2006). Multipotent mesenchymal stem cells reduce interstitial fibrosis but do not delay progression of chronic kidney disease in collagen4A3-deficient mice. Kidney Int.

[b28] Prodromidi EI, Poulsom R, Jeffery R (2006). Bone marrow-derived cells contribute to podocyte regeneration and amelioration of renal disease in a mouse model of Alport syndrome. Stem Cells.

[b29] Held PK, Al-Dhalimy M, Willenbring H (2006). *In vivo* genetic selection of renal proximal tubules. Mol Ther.

[b30] Li L, Truong P, Igarashi P (2007). Renal and bone marrow cells fuse after renal ischemic injury. J Am Soc Nephrol.

[b31] Togel F, Hu Z, Weiss K (2005). Administered mesenchymal stem cells protect against ischemic acute renal failure through differentiation-independent mechanisms. Am J Physiol Renal Physiol.

[b32] Yeagy BA, Harrison F, Gubler MC (2011). Kidney preservation by bone marrow cell transplantation in hereditary nephropathy. Kidney Int.

[b33] Valerius T, Elsasser D, Repp R (1997). HLA class II antibodies recruit G-CSF activated neutrophils for treatment of B cell malignancies. Leuk Lymphoma.

[b34] Garcia-Bravo M, Moran-Jimenez MJ, Quintana-Bustamante O (2009). Bone marrow-derived cells promote liver regeneration in mice with erythropoietic protoporphyria. Transplantation.

[b35] Togel F, Isaac J, Westenfelder C (2004). Hematopoietic stem cell mobilization-associated granulocytosis severely worsens acute renal failure. J Am Soc Nephrol.

[b36] Humphreys BD, Valerius MT, Kobayashi A (2008). Intrinsic epithelial cells repair the kidney after injury. Cell Stem Cell.

[b37] Tamama K, Fan VH, Griffith LG (2006). Epidermal growth factor as a candidate for *ex vivo* expansion of bone marrow-derived mesenchymal stem cells. Stem Cells.

[b38] Rowinsky EK, Schwartz GH, Gollob JA (2004). Safety, pharmacokinetics, and activity of ABX-EGF, a fully human anti-epidermal growth factor receptor monoclonal antibody in patients with metastatic renal cell cancer. J Clin Oncol.

[b39] Petrides PE, Bock S, Bovens J (1990). Modulation of pro-epidermal growth factor, pro-transforming growth factor alpha and epidermal growth factor receptor gene expression in human renal carcinomas. Cancer Res.

[b40] Price JT, Wilson HM, Haites NE (1996). Epidermal growth factor (EGF) increases the *in vitro* invasion, motility and adhesion interactions of the primary renal carcinoma cell line, A704. Eur J Cancer.

[b41] Bashir O, Fitzgerald AJ, Berlanga-Acosta J (2003). Effect of epidermal growth factor administration on intestinal cell proliferation, crypt fission and polyp formation in multiple intestinal neoplasia (Min) mice. Clin Sci.

