# Supplementary figures and images for: Epidermal growth factor attenuates tubular necrosis following mercuric chloride damage by regeneration of indigenous, not bone marrow-derived cells
Source: J Cell Mol Med. 2014 Nov 11;19(2):463–73. doi: 10.1111/jcmm.12478 (PMC4407604; doi:10.1111/jcmm.12478)

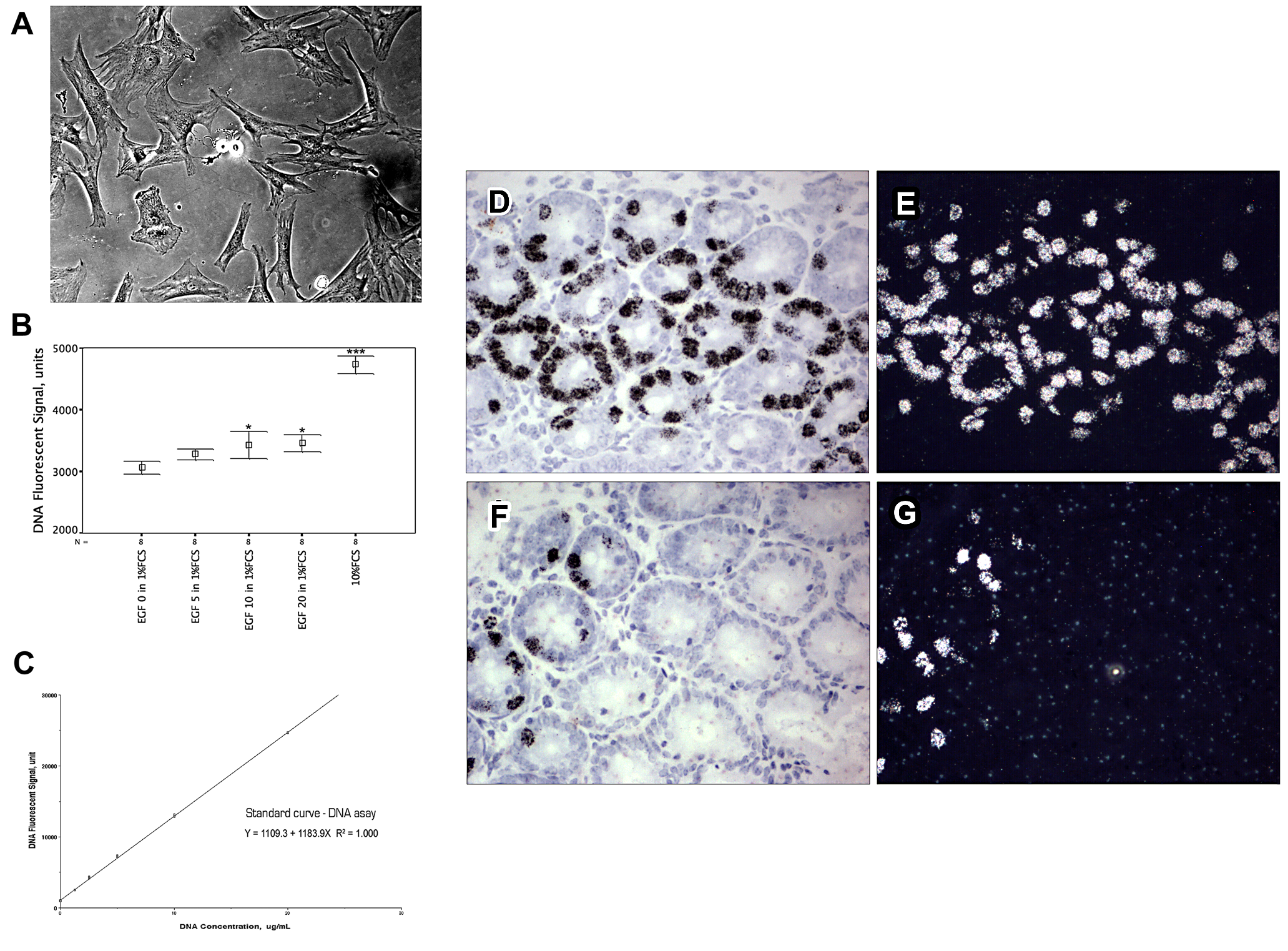

Supplement: Supplementary file 1 [file jcmm0019-0463-sd1.tif]
